# Supplementary material for: Effects of genetic variability of CYP2D6 on neural substrates of sustained attention during on-task activity
Source: Transl Psychiatry. 2020 Oct 6;10:338. doi: 10.1038/s41398-020-01020-z (PMC7539151; doi:10.1038/s41398-020-01020-z)
Supplement: Supplementary file 1 — Supplemental Material [file 41398_2020_1020_MOESM1_ESM.doc]

# Supplementary material

Table 1s: Primers used for determination of CYP2D6 CNV

| Primer | CNV | Fragment size |
| --- | --- | --- |
| **CYP2D6-Forward**  CCAGAAGGCTTTGCAGGCTTCA  **CYP2D6-Reverse**  ACTGAGCCCTGGGAGGTAGGTA | no | 5.1kb |
| **CYP-207-Forward**  CCCTCAGCCTCGTCACCTCAC  **CYP-32-Reverse**  CACGTGCAGGGCACCTAGAT | Duplication specific fragment | 3.2 kb |
| **CYP-13-Forward**  ACCGGGCACCTGTACTCCTCA  **CYP-32-Reverse**  CACGTGCAGGGCACCTAGAT | Duplication internal control | 3.8 kb |
| **CYP-13-Forward**  ACCGGGCACCTGTACTCCTCA  **CYP-24-Reverse**  GCATGAGCTAAGGCACCCAGAC | Deletion specific fragment | 3.5 kb |
| **CYP-207-Forward**  CCCTCAGCCTCGTCACCTCAC  **CYP-24-Reverse**  GCATGAGCTAAGGCACCCAGAC | Deletion internal control | 3.0 kb |

Table 2s. Results of genotyping

| Number of subjects | Activity score | Age (average) | Age (std. dev.) | Number of females |
| --- | --- | --- | --- | --- |
| 22 | 0 | 24.2 | 5.4 | 13 |
| 27 | 0.5 | 22.8 | 3.4 | 16 |
| 122 | 1 | 23.3 | 3.9 | 70 |
| 54 | 1.5 | 24.2 | 4.4 | 31 |
| 181 | 2 | 23.3 | 3.3 | 100 |
| 9 | 3 | 24.3 | 3.5 | 5 |
